# Structural Health Monitoring of Fiber Reinforced Composites Using Integrated a Linear Capacitance Based Sensor

**DOI:** 10.3390/polym16111560

**Published:** 2024-05-31

**Authors:** Khalid S. Alblalaihid, Saad A. Aldoihi, Abdulaziz A. Alharbi

**Affiliations:** 1 Institute of Space and Earth Science, King Abdulaziz City for Science and Technology (KACST), Riyadh 11442, Saudi Arabia; alblehed@kacst.gov.sa; 2 Institute of Advanced Material, King Abdulaziz City for Science and Technology (KACST), Riyadh 11442, Saudi Arabia

**Keywords:** coating, capacitive sensor, fiber-reinforced polymer composites (FRPC), structural health monitoring (SHM), physical vapor deposition, smart material

## Abstract

The demand for fiber-reinforced polymers (FRPs) has significantly increased in various industries due to their attributes, including low weight, high strength, corrosion resistance, and cost-efficiency. Nevertheless, FRPs, such as glass and Kevlar fiber composites, exhibit anisotropic properties and relatively low interlaminar strength, rendering them susceptible to undetected damage. The integration of real-time damage detection processes can effectively mitigate this issue. This paper introduces a novel method for fabricating embedded capacitive sensors within FRPs using a coating technique. The study encompasses two types of fibers, namely glass and Kevlar fiber/epoxy composites. The physical vapor deposition (PVD) technique is employed to coat bundle fibers with conductive material, thus creating embedded electrodes. The results demonstrate the uniform distribution of nanoparticles of gold (Au) along the fibers using PVD, resulting in a favorable resistance of approximately ≈100 Ω. Two sensor configurations are explored: axial and lateral embedding of the coated yarn (electrodes) to investigate the influence of load direction on the coating yarn. Axial-sensor configuration specimens undergo tensile testing, showcasing a linear response to axial loads with average sensitivities of 1 for glass and 1.5 for Kevlar fiber/epoxy composites. Additionally, onset damage is detected in both types of fiber composites, occurring before final fracture, with average stress at the turning point measuring 208 MPa for glass and 144 MPa for Kevlar. The lateral-sensor configuration for glass fiber-reinforced polymer (GFRP) exhibits good linearity towards strain until failure, with average gauge factors of 0.25 and −2.44 in the x and y axes, respectively.

## 1. Introduction

The demand for fiber-reinforced polymer (FRP) composites has been steadily increasing in various structural materials applications [1]. For instance, the use of glass fiber/epoxy composites in the construction of unmanned aerial vehicles (UAVs) highlights the appeal of FRP composites due to their chemical resistance, flexibility, and high strength-to-weight ratio. However, FRPs are characterized by their heterogeneity and relatively low interlaminar strength, making them prone to mechanical property degradation. This degradation can arise from unexpected overloads, matrix cracking, fiber fragmentation, and delamination between layers, often occurring without prior warning. Consequently, regular inspections using non-destructive testing (NDT) are essential to ensure structural integrity and operational safety in FRP composite structures. However, periodic inspections can lead to delayed detection of induced damage, resulting in increased downtime costs. Therefore, the implementation of real-time, in situ damage detection systems holds paramount importance in anticipating defects and mitigating this problem [2].

A multitude of methods for structural health monitoring (SHM) have been developed, with ongoing research efforts [3]. Some approaches involve the installation of additional sensors into composite structures, such as optical sensors [4] and piezoelectric-based acoustic emission sensors [5]. This work focuses on the development of multifunctional materials, with a particular emphasis on self-sensing capabilities. Self-sensing materials possess the unique ability to detect deformations and damages without the need for supplementary sensors, offering substantial advantages in terms of reduced labor, weight, and cost savings [6]. One approach to achieving these capabilities is by integrating network sensors with epoxy matrices [7]. The literature showcases a variety of techniques for developing multifunctional materials to ensure the integrity of composite structures [8].

Recent advancements include the development of voltage sensors based on coating fiber fabric with piezoelectric materials using dip techniques [9]. In these approaches, fiber weaves are immersed in microparticles of piezoelectric barium titanate (BaTiO_3_) mixed with epoxy to form the sensor. However, conventional piezo-resistive sensors are primarily suited for measuring strain and detecting damage in conductive fibers, such as carbon fiber-reinforced polymers (CFRPs) [10]. Fibers like glass and Kevlar, being non-conductive materials, present challenges in integrating piezo-resistive sensors effectively. To enhance electrical conductivity for SHM, nanoparticles, such as carbon nanotubes (CNTs), are typically dispersed within an epoxy resin to modify the electrical connectivity of the polymer composite matrix [11,12,13]. Even though the nanomaterials merged with the matrix have a high strain gauge factor, the main load in the composite is carried by fiber reinforcement, which can lead to limited information [14]. Additionally, this approach can lead to electromagnetic shielding for FRPs, potentially interfering with signal transmission between onboard and ground systems, particularly in critical applications like UAVs where telemetry and autopilot data are paramount.

The integration of electrically conductive materials at the level of fiber yarn offers the potential to transmit electromagnetic signals through the structure and provide direct strain measurements as the fiber yarn carries the primary load. Recent interest in enhancing the functionality of fiber yarns has grown, with one significant development being the coating of these yarns with conductive materials to enable real-time piezo-resistive sensing of structural responses [15]. Several methods for applying conductive coatings to fiber yarns have been explored, including chemical vapor deposition (CVD) [16], electrodeposition [17], dip-coating [18], and spray-coating [19]. Another form of the recently developed piezoresistive sensor is that nylon yarn was coated with gold nanoparticles using an electroless plating technique and integrated into composite specimens. The gauge factor was measured to be within a range of 21–25 [20]. However, resistance-based sensors face challenges, including resistance accumulation due to small cracks, electrical tunneling effects [14], and the need for complex and expensive four-probe methods [21].

An alternative technique employed in FRP materials is the use of capacitance sensors, typically consisting of two electrodes separated by a dielectric material. Studies have shown that the carbon fiber used as a reinforcing material in epoxy resin has the capability to be electrically conductive and can function as electrodes, which can be separated by a dielectric material such as paper to form a supercapacitor [22]. Supercapacitors were also developed by using GFRP composite as dielectric and metalized film used as electrodes [23]. Capacitance sensors can also function as strain sensors in FRP composites [24]. When subjected to external forces, these sensors deform in various dimensions (thickness of the dielectric, width, and length of electrodes), resulting in a change in capacitance value, effectively serving as strain sensors. Yan et al. [24] introduced a multifunctional CFRP incorporating a capacitive sensor. This design incorporated a dielectric layer made of titania-filled epoxy, sandwiched between two cured CFRP laminates, exhibiting a static gauge factor of approximately 0.92. Furthermore, the capacitance sensor can be utilized to detect damage and moisture content in FRP structures, with changes in the dielectric properties signaling induced interfacial damages and moisture penetration [25]. Shen et al. [26] investigated the impact of axial stress on the structural capacitor of CFRP, experimenting with three different dielectric materials: polyethylene terephthalate (PET), trilayer polypropylene–polyethylene–polypropylene (PP-PET-PP), and cis-polyisoprene (latex). Their research revealed that the structural capacitor deteriorated during the early stages of axial loading, coinciding with the initiation of interfacial cracking between the dielectric layers and CFRP. Recently, Buggisch et al. [27,28] developed an analytical model that establishes a correlation between interfacial damage and a reduction in the embedded capacitance value. They embedded interleaved capacitors into GFRP (glass fiber-reinforced polymer), employing three types of electrodes, including carbon fiber yarn, narrow copper wires, and solid copper wires. Their findings indicated that damage occurred at an early stage of axial stress. In a study by Alblalaihid et al. [29,30], a smart intra-ply hybrid composite material (comprising glass fiber and carbon fiber) based on capacitance was developed. It was observed that failure occurred at a low level of axial stress due to the combination of low-elongation and high-elongation fibers within a common matrix.

To demonstrate the feasibility of embedded capacitive sensing electrodes for strain monitoring and early-stage damage detection in woven fabric composites, this study employs a coating technique rather than integrating new and foreign conductive fibers into the matrix to prevent early-stage failure occurring at a low level of axial stress. Two types of fibers, glass and Kevlar, are considered for the coating process, utilizing physical vapor deposition (PVD). The study examines the structural features of the two materials using Scanning Electron Microscopy (SEM) and conducts tensile tests to characterize the electromechanical properties and validate self-sensing functionality in the smart structure. The overarching objective of this work is to develop embedded capacitive sensing electrodes that enable strain monitoring within a linear range and facilitate the early detection of damage in woven fabric composites.

## 2. Materials and Methods

### 2.1. Working Principle of the Embedded Capacitive Sensor into FRP

**Figure 1:**
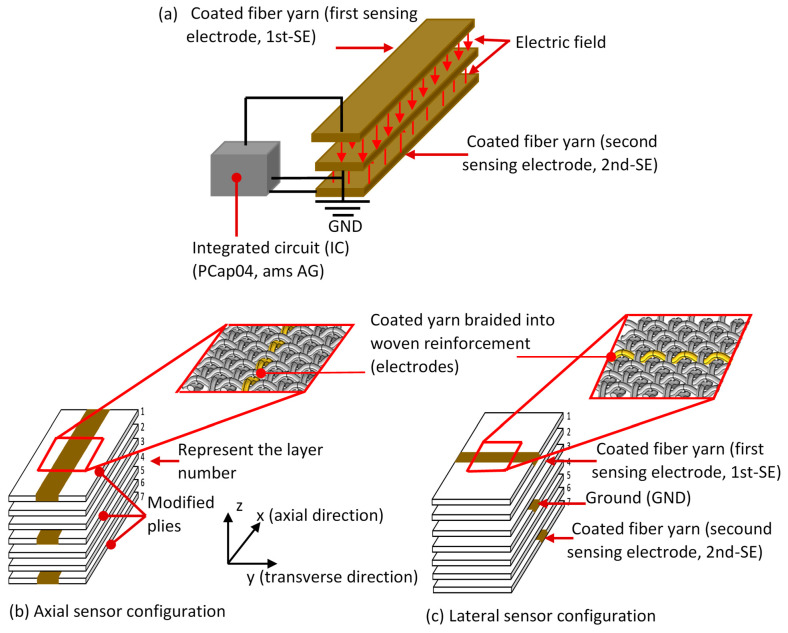
(**a**) Schematic representation of dual parallel plate capacitors, which is analogous to the embedded capacitance concept within FRP. The layup profile is employed to create the embedded capacitive sensor in (**b**) the axial (*x*-axis) and (**c**) lateral (transverse) directions (*y*-axis).

The proposed self-sensing fiber-reinforced polymer composites are based on a dual differential capacitive sensor, as illustrated in Figure 1a. The capacitive sensor consists of a three-electrode system embedded in plies 1, 4, and 7, referred to as modified plies. The electrode in the middle ply (number 4) was connected to the ground signal (GND), while ply numbers 1 and 7 were connected to the first (1st-SE) and second sensing electrode (2nd-SE), respectively, forming dual differential capacitive sensors. Hence, two sensors were implemented in the FRP composite, with the first sensing electrode (1st-SE) monitoring plies number 1 to 4 and the second sensing electrode (2nd-SE) monitoring plies number 4 to 7. These two electrodes were separated by an unmodified ply (woven fabric), serving as the dielectric material.

To investigate the detection of the onset of tensile failure and the strain sensitivity of the embedded sensor concerning the direction of applied load (axial and transverse), two different capacitive sensor configurations were considered. The coated filament yarns forming electrodes in the modified plies were integrated along the *x*-axis (axial-sensor configuration) to measure the sensitivity of the embedded capacitive sensor to forces applied along the *x*-axis (see Figure 1b). To characterize the performance of the capacitive sensor in response to perpendicular loads (lateral-sensor configuration), the coated filament yarns (electrodes) were braided along the *y*-axis, as illustrated in Figure 1c. The gauge factors in the *x*-axis (*k_x_*) and *y*-axis (*k_y_*) were measured using Equations (1) and (2) to assess the strain sensitivity for both embedded capacitive sensor configurations [24].

(1) $$k_{x}=\frac{\frac{∆C}{C_{O}}}{\varepsilon_{x}}$$

(2) $$k_{y}=\frac{\frac{∆C}{C_{O}}}{\varepsilon_{y}}$$

here:

- ∆C is the incremental change in capacitance value,
- $C_{O}$ is the initial measured capacitance,
- $\varepsilon_{x}$ and $\varepsilon_{y}$ are the measured strains in the axial and lateral directions obtained using strain gauge sensors.

### 2.2. Preparation of Coated Fibres Yarn (Electrodes) for Capacitive Sensing

To create an embedded capacitive sensor for strain monitoring in fiber-reinforced polymer (FRP), filament yarns were coated to form electrodes. Two different materials of filament yarn, namely glass and Kevlar fiber, were considered in this investigation. Various conductive coating techniques were evaluated, and physical vapor deposition (PVD) was selected as the appropriate coating technique, based on previous work [31]. PVD was chosen for its lower operational temperatures, which are vital for avoiding thermal degradation of the fibers and ensuring their continued structural and functional integrity [32]. Additionally, PVD’s capability to apply coatings that are both thinner and more precise is crucial for this application [33]. Such precision is necessary to avoid full coverage of the fibers, preventing agglutination that could impede resin penetration and consequently diminish the strength of the composite material.

**Figure 2:**
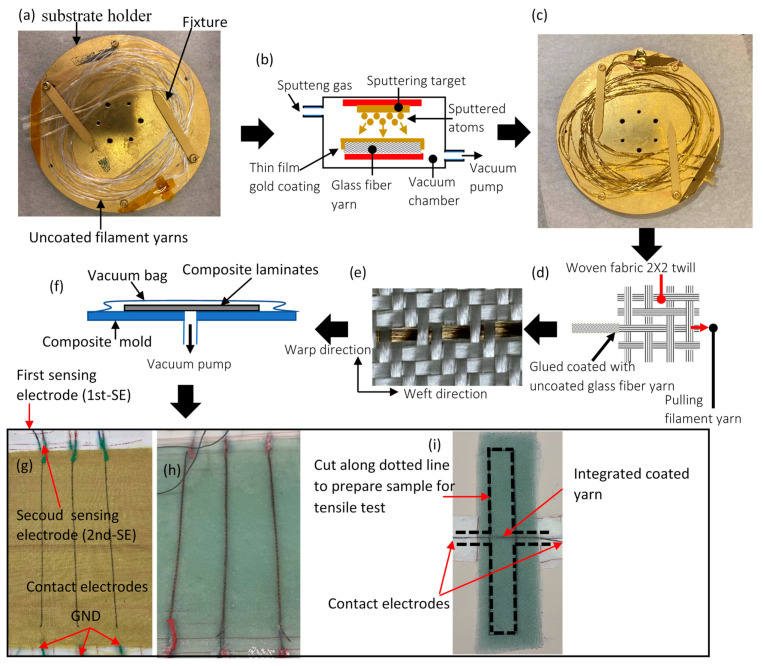
Fabrication process of FRP embedded sensor: (**a**) A photograph illustrating the procedure for positioning filament yarns on the substrate holder before the coating process. (**b**) A schematic diagram depicting the basic setup for physical vapor deposition (PVD). (**c**) An image displaying the final shape of coated filament fiber yarns. (**d**) A schematic representation of the integration process for coated filament yarn into woven fabric. (**e**) An image showcasing the integration of coated glass fiber yarn into the woven fabric via PVD coatings. (**f**) Vacuum bagging system. (**g**,**h**) Images of cured laminates for axial sensor configuration with coated Kevlar and glass fiber yarn. (**i**) Images of cured laminates for lateral sensor configuration with coated glass fiber yarn.

Figure 2 illustrates the fabrication process used to create smart Kevlar (Plain, P170A, hp-textiles GmbH, Schapen, Germany) and fiberglass (2 × 2 twill, FK 144, Interglas-92125, Haufler Composites, Blaubeuren, Germany) reinforced composite structures with embedded differential capacitive sensors. To simultaneously coat several filament yarns (Kevlar or glass fiber) extracted from the weft direction, the yarns were spirally wound on a substrate holder, as shown in Figure 2a. Subsequently, the substrate holder was inserted into the PVD apparatus for the deposition of electrically conductive nanoparticles on the filament yarns’ surface. This deposition was achieved using an ATC-Orion 8 sputtering system, which employs magnetron sputtering to deposit thin films of gold (Au) onto the substrate, as depicted in the schematic diagram in Figure 2b. Gold (Au) was selected as the coating material due to its superior corrosion and oxidation resistance, ensuring stability and longevity in conductive connections [34]. To enhance coating adhesion and mechanical reliability, gold (Au) was co-deposited with copper (Cu) at growth rates of 0.46 nm/s and 0.1 nm/s, respectively [35]. The substrate holder was motorized, allowing for a rotating platform to achieve excellent coating uniformity and the formation of multilayer thin film metal within nanometer thickness. The filament yarns remained in the vacuum chamber for approximately 22 min during the coating process, with the following settings: a chamber pressure of 6 × 10^−3^ Torr and a deposition power of 100 W. To ensure uniform coating on all surfaces, the yarns were flipped upside down after the initial deposition, and the process was repeated as described above.

**Figure 3:**
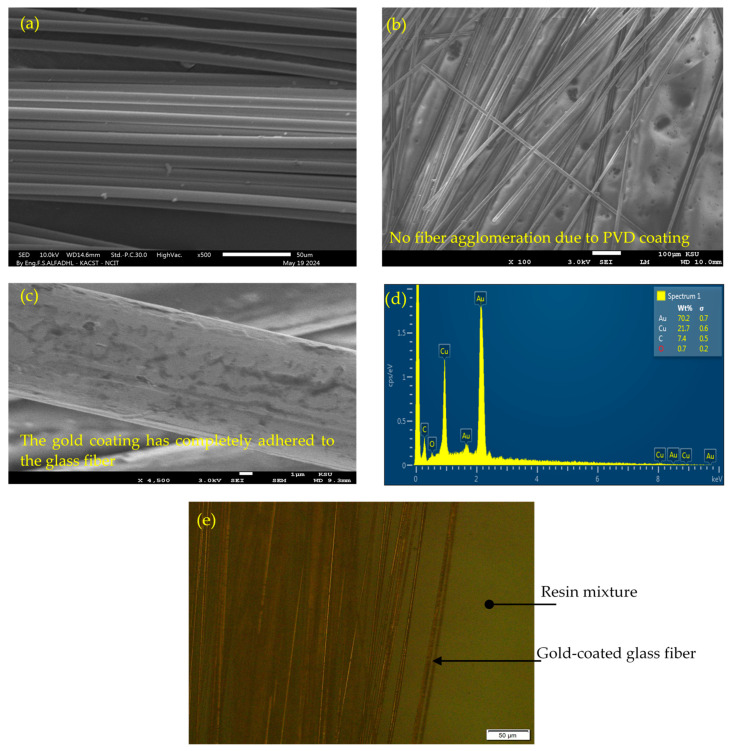
(**a**,**b**) SEM images of uncoated and PVD-coated glass fiber yarn, respectively. (**c**) Micrographs of individual filaments coated with PVD. (**d**) Elemental analysis (EDX) of the PVD-coated glass fiber. (**e**) Microscopic view of a gold-coated glass fiber yarn filament embedded in an epoxy resin matrix after curing.

**Figure 4:**
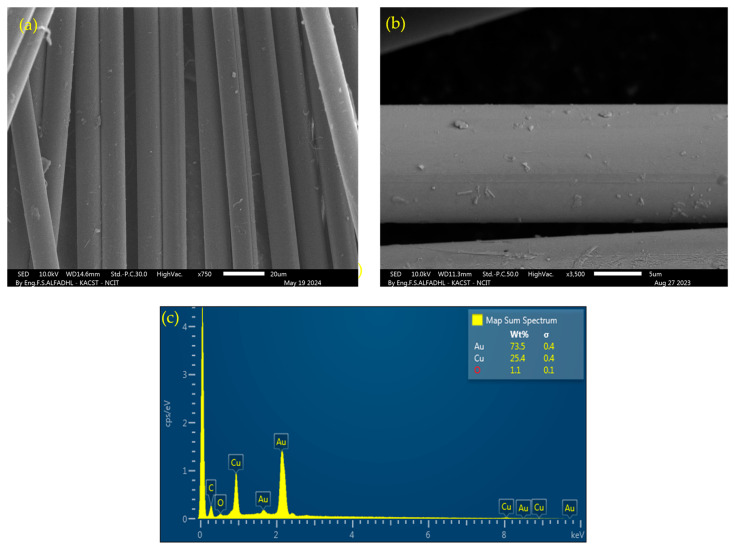
(**a**,**b**) SEM images of uncoated Kevlar fiber yarn and a single PVD-coated Kevlar fiber, respectively. (**c**) Elemental analysis (EDX) of the PVD-coated Kevlar fiber.

Figure 2c displays the final shape of four coated filament yarns, each 1 m in length, folded into a spiral shape and placed on the substrate. PVD offers the advantage of producing metallic (Au) nano-multilayers with a uniform surface morphology. To assess this capability, the structural features of the coated yarns were visually inspected using Scanning Electron Microscopy (SEM) (JOEL JSM-7600F, FE-SEM, Tokyo, Japan) with an accelerating voltage ranging from 3–5 kV. Figure 3a,b display the typical morphologies of uncoated and coated fiber yarn, respectively. Notably, there were no unwanted fiber agglomerates caused by the PVD coating, which would result in single fibers adhering to adjacent fibers, as shown in Figure 3b. To demonstrate the morphologies of virgin Kevlar fiber and coated Kevlar fiber, SEM images were captured and are presented in Figure 4a and Figure 4b, respectively.

The coating layer was theoretically composed of 80% Au and 20% Cu, based on the coating parameters of growth rate and deposition time. To verify the composition of the deposition coating on both surface-coated glass and Kevlar fibers, Energy Dispersive X-ray Spectroscopy (EDX) analysis was conducted. The analysis confirmed the composition percentages of the coating for both glass and Kevlar fibers, with the glass fiber consisting of 70% Au and 21% Cu and the Kevlar fiber consisting of 73% Au and 25% Cu, as shown in Figure 3d and Figure 4c.

The integration process, described in Figure 2d, was also used to braid the coated yarn into the woven fabric. Figure 2e illustrates the final shape of the integrated coated glass fiber yarn into the woven fabric via PVD coating. The epoxy resin (L285, HEXION, Stuttgart, Germany) was mixed with hardener (H287, HEXION, Stuttgart, Germany) at room temperature, with a weight ratio of 100/40. To fabricate Kevlar and glass fiber-reinforced polymer composites with modified plies, seven layers were stacked and impregnated with the resin mixture under vacuum to remove bubbles, as shown in the schematic diagram of Figure 2f. Ensuring that the reinforcement material saturates with the liquid resin system increases the clarity of coated fiber yarns between plies, facilitating their alignment on top of each other to form the differential capacitance sensors. The final shape of the cured laminate for Kevlar and glass fiber-reinforced polymer composites, with electrodes configured in the axial direction, is illustrated in Figure 2g and Figure 2h, respectively. Additionally, Figure 2i displays images of cured laminates with lateral sensor configurations for glass fiber-reinforced polymer composites. To verify that the problems encountered in prior studies [31], specifically the dispersion of spray carbon coating within the epoxy resin, were not repeated, a gold-coated glass fiber yarn filament embedded in an epoxy resin matrix underwent visual inspection via microscopy post-curing, as depicted in Figure 3e. This examination, carried out using an OLYMPUS MX61L microscope, (Tokyo, Japan), confirmed that the gold coating remained intact without dispersal. The microscope was calibrated to micrometers (µm), a scale chosen in consideration of the coated layer’s estimated thickness of approximately 0.6 µm, derived from the deposition rate and coating time. This setting ensured that any potential spread of the coating would be clearly visible within the anticipated total thickness of 0.6 µm.

### 2.3. Experimental Setup

The embedded dual differential capacitance sensors (1st-SE and 2nd-SE) in Kevlar and glass fiber-reinforced polymer composites can be employed to sense various physical quantities. However, the scope of this work is to characterize the capacitive sensor for measuring deformation when the structure is subjected to axial forces and detecting the onset of damage between layers.

To initially characterize the electromechanical behavior of the self-sensing for both sensor configurations (axial and lateral), a tensile test was performed. For both sensor configurations, samples were cut according to ASTM D3039 [36] using an oscillating cutting tool. Regarding the lateral sample, the cutting was made along a dotted line to allow electrical connection for the capacitive sensor, as shown in Figure 2i.

To protect the tensile test pieces from damage by the wedge grips and to provide electrical protection for the coated fiber yarns (electrodes), glass/epoxy tabs, each 40 mm long, were adhered to the ASTM D3039 specimens. The final geometric parameters of the specimens are presented in Table 1. It is crucial to confirm that the embedded sensor provides reliable experimental measurements under external stress conditions. As a result, two samples were prepared for each type to conduct the electromechanical test. 

**Figure 5:**
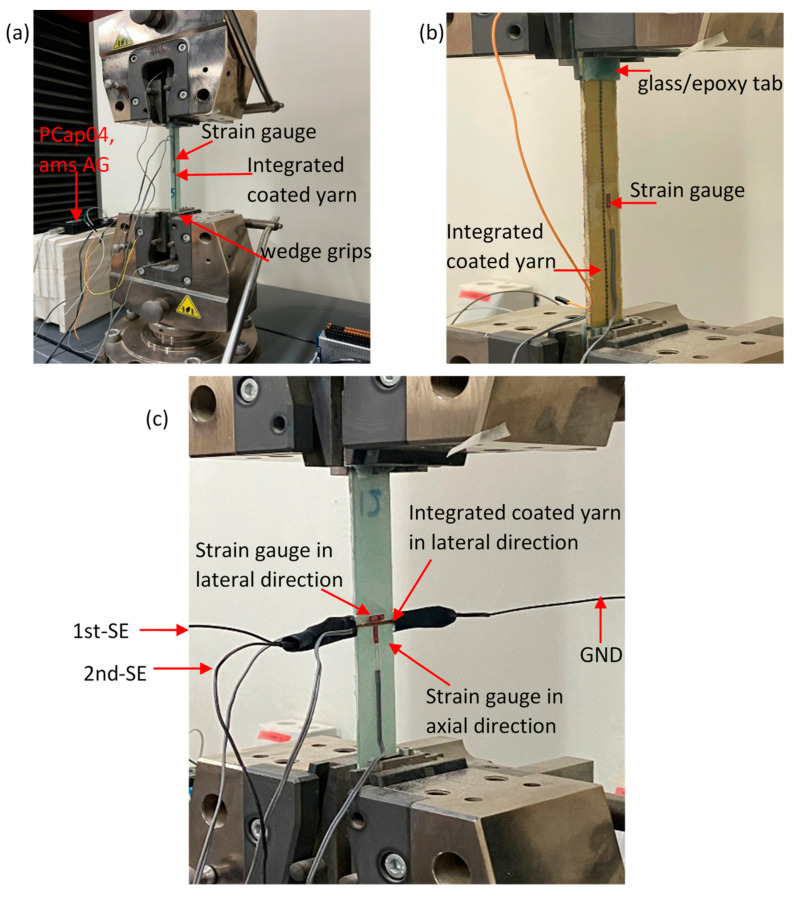
Photographs of the experimental arrangement employed for conducting the tensile test: (**a**) for the axial-sensor configuration using coated glass fiber yarn and (**b**) for the axial-sensor configuration using coated Kevlar aramid fiber yarns. (**c**) An image of the lateral-sensor configuration with coated glass fiber yarn being loaded onto the tensile test machine.

A universal tensile test machine (Zick/Roell, Z100, Ulm, Germany) with a load cell of 100 kN was used to measure the axial tensile force exerted on the samples at a crosshead rate of 1 mm/min. A strain gauge sensor (Measuring Instruments Laboratory, Tokyo, Japan, FLA5-11-1L) was also used to evaluate the gauge factor for the embedded differential capacitance sensor and estimate Young’s modulus of the specimens. This transducer was glued at the mid-gauge length of specimens, as seen in Figure 5. An additional strain gauge sensor was mounted on the lateral sensor configuration samples in the lateral direction to measure the strain in width as the sample was loaded. The sampling rate of the axial force and strain, acquired by the tensile test machine and National Instruments hardware (NI cDAQ-9172, NI-9237, Austin, TX, USA), was 50 Hz. To measure the relative change in dual differential capacitance, three-wire sensors were connected to a commercially available integrated circuit (PCap04, ams AG, Premstätten, Austria) with a sampling rate of 6 Hz. Analog signals of the strain, force, and capacitance were recorded simultaneously during the test to accurately evaluate the electromechanical behavior of the embedded sensors.

## 3. Result and Discussion

### 3.1. Surface Morphology and Coating Adhesion

The surface morphologies of the coated yarn and single filament with an electrically conductive layer over glass and Kevlar fiber yarn are presented in Figure 3 and Figure 4. It is evident that the PVD coating process did not result in any unwanted fiber agglomerates. The absence of agglomeration is crucial as it ensures the flexibility of the yarn, which is essential for its integration into woven fabric structures. Furthermore, the absence of agglomeration prevents hindrance to the penetration and impregnation of epoxy resin into the coated fibers during the layup process. This is significant as incomplete impregnation can have a substantial impact on the mechanical properties of the resulting composite.

The compatibility of the coated fibers with epoxy resin was visually inspected, and it was observed that the coating did not disperse into the polymer matrix during the curing process as illustrated in Figure 3e. This suggests strong adhesion between the coating and the fibers. Additionally, the resistance of the coated yarn was found to be on the order of a few hundred ohms for a 1 m length, which is considered to be a fairly low resistance suitable for forming electrodes. In the following section, the 2D in-plane strain sensing capabilities of embedded sensors in both glass fiber and Kevlar-reinforced polymer were assessed using two sensor configurations, namely axial and lateral.

### 3.2. Sensor Performance in Axial Direction

**Figure 6:**
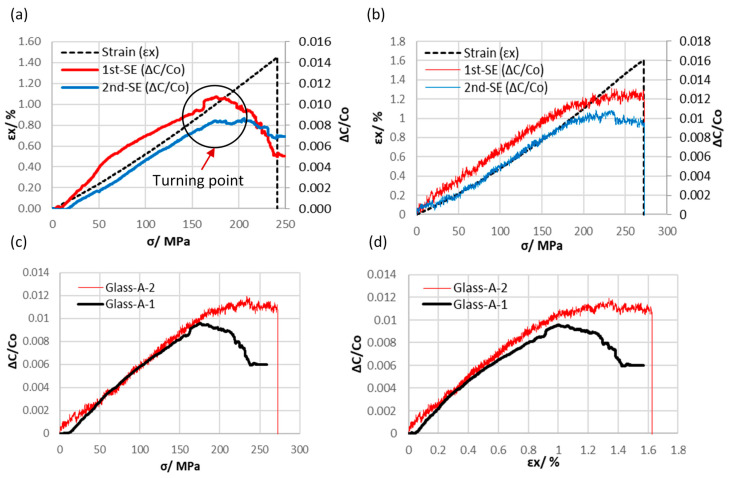
A plot illustrating the relative change in capacitances (first and second sensing electrodes) and nominal strain versus stress for axial sensor configuration specimens of (**a**) Glass-A-1, (**b**) Glass-A-2. (**c**) Axial stress and (**d**) strain curves against the average capacitance value of the first and second sensing electrodes for GFRP specimens.

**Figure 7:**
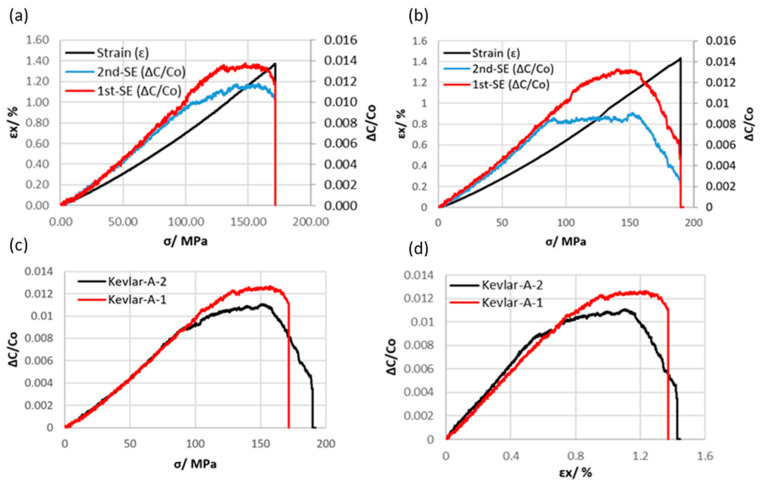
A plot depicting the relative capacitance change (first and second sensing electrodes) and nominal strain in relation to stress for axial sensor configuration specimens of (**a**) Kevlar-A-1 and (**b**) Kevlar-A-2. (**c**) Axial stress and (**d**) strain curves plotted against the average capacitance values of the first and second sensing electrodes for Kevlar-reinforced polymer epoxy composite specimens.

To complete the characterization of samples with axial sensor configuration (Glass-A and Kevlar-A), quasi-static tensile tests were conducted. These tests involved loading the samples into a tensile machine, as shown in Figure 5a,b, with axial loads carried by the coated fiber yarns oriented along the weft direction. Stress–strain and relative capacitance change data were recorded for each sample until the point of failure. The results for Glass-A and Kevlar-A samples are illustrated in Figure 6 and Figure 7, respectively. It can be observed that all samples exhibited a linear stress–strain relationship up to the point of failure. The Young’s modulus for all specimens was determined by dividing the maximum axial stress by the maximum strain at the fracture point, and the results are outlined in Table 2. To facilitate a comparison between the original and altered glass fiber/epoxy composite, we used the mechanical properties of GFRP obtained from testing specimens in the lateral sensor configuration (as detailed in Section 3.3) as the reference baseline. This could be attributed to the integrated coated yarn in the lateral direction, where its influence on mechanical properties is minimal since the coated yarn is not subjected to direct loading. When comparing the mechanical properties of the lateral sensor configuration specimens in GFRP (see Table 3) with those of the axial sensor configuration (Glass-A, see Table 2), it is evident that the differences in Young’s modulus and ultimate strength were less than 6%. This suggests that the integration of the coated yarn as a sensor did not significantly affect the mechanical properties of the composite. 

To evaluate the influence of the integrated capacitive sensor on the mechanical properties of the Kevlar fiber/epoxy composite, a pristine sample was created and subjected to tensile testing. This pristine specimen had a thickness of 2.31 mm and a width of 25.6 mm, yielding Young’s modulus and ultimate strength measurements of 14 GPa and 249 MPa, respectively. For the Kevlar fiber-reinforced polymer composite (Kevlar-A), a notable difference in ultimate strength (approximately 27%) was observed in comparison to the pristine specimen. The Kevlar-A samples, labeled as Kevlar-A-1 and Kevlar-A-2, had thicknesses of 2.9 mm and 2.68 mm (as indicated in Table 1), which were thicker than the pristine sample (2.31 mm). This variation may be attributed to the increased thickness of the Kevlar-A samples, resulting from the manufacturing process, potentially impacting the pressure during vacuum bagging [26] and introducing residual stresses during curing [37].

The baseline capacitance (Co) for both the first and second sensing electrodes (1st-SE and 2nd-SE) was averaged for each sample, and the results are provided in Table 2. These measurements were conducted prior to subjecting the samples to any axial loads. To characterize the embedded differential capacitive sensors in GFRP composites (Glass-A-1 and Glass-A-2), the relationship between the output signals of the 1st-SE and 2nd-SE for each sample was plotted as a function of axial stress. The relative capacitance change of these electrodes was found to be linearly proportional to the axial stress and strain rate, as shown in Figure 6a,b. It is clear that there is a slight variation in the relative capacitance change between the 1st-SE and 2nd-SE during testing for both specimens, Glass-A-1 and Glass-A-2. This deviation may be attributed to instances of off-center loading during the tensile test, which can consequently generate small, undesired bending moments. However, Figure 6c displays a graph illustrating the average capacitive sensor outputs in relation to axial stress for Glass-A-1, which closely aligns with the graph for the Glass-A-2 specimen. The relative capacitance change curve exhibited a deviation from linear increase at a turning point for both Glass-A-1 and Glass-A-2 specimens. This turning point may indicate the onset of inter-fiber damage, where the relative capacitance change decreases or remains constant while the axial stress increases [27]. The average relative capacitance change, strain, and axial stress at the turning point were approximately 1%, 1.18%, and 208 MPa, respectively, as summarized in Table 2. However, it has been observed that the strain rate of the embedded capacitive sensor is linear within a wide strain range, covering approximately 70% of the total strain range. This suggests that the coated yarn did not cause early-stage damage to the glass fiber-reinforced polymer, unlike previous research where carbon fiber yarn was used as an electrode and integrated into glass fiber composites. This indicates that the current approach is less detrimental to the composite’s integrity [29]. The gauge factor, computed using a linear least squares method over a 0.8% strain range for the embedded capacitive sensors according to Equation (1), was approximately 1.

The embedded capacitive sensors in Kevlar fiber-reinforced polymer composites were also examined. The relative capacitance change for both Kevlar-A-1 and Kevlar-A-2 samples increased linearly up to a turning point, equivalent to 75% of the total strain range as shown in Figure 7a,b. The average gauge factor was estimated to be 1.4, which is relatively higher than the gauge factor for the embedded sensor in GFRP. The onset of damage was detected at the turning point, where the growth of matrix cracks before final failure led to a decrease in capacitance.

**Figure 8:**
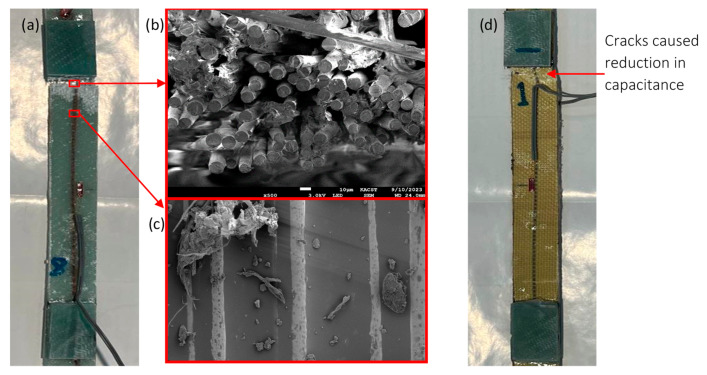
(**a**) A photograph depicting a typical fracture in a glass fiber/epoxy composite specimen. (**b**) SEM image illustrating the breakage of coated glass fiber and the subsequent debonding from the polymer matrix at the failure zone. (**c**) SEM image capturing a close-up view of the coated glass fiber surface near the fracture zone. (**d**) A photograph showcasing a typical fracture in a Kevlar fiber/epoxy composite specimen.

Post-failure inspections of specimens were carried out using scanning electron microscopy (SEM) to investigate failure modes in the vicinity of the coated yarn. In Figure 8a,d, the final failures of the integrated sensor systems within the glass and Kevlar fiber/epoxy composite specimens are depicted, occurring near the tab zone. The initiation of failure, detected by the capacitive sensor at the turning point, was linked to damage in the longitudinal yarn (coated yarn), followed by fiber fracture, resulting in average ultimate strains of 1.52% and 1.41% for Glass-A and Kevlar-A specimens, respectively. Microstructural damage in the integrated coated glass fiber within GFRP was examined using SEM, as shown in Figure 8b. This revealed coated fiber pull-out and debonding from the polymer matrix. SEM images of the coated glass fiber near the fracture zone indicated that the surface remained intact and well adhered to the polymer matrix, as demonstrated in Figure 8c.

### 3.3. Sensor Performance in the Lateral Direction

The evaluation of lateral (transverse) strain sensing performance was carried out only for glass fiber/epoxy composite with the lateral sensor configuration (Glass-L). The setup included strain gauges adhered in both axial and lateral directions to record strain (*ɛ_x_* and *ɛ_y_*) up to the fracture point, as shown in Figure 5c.

**Figure 9:**
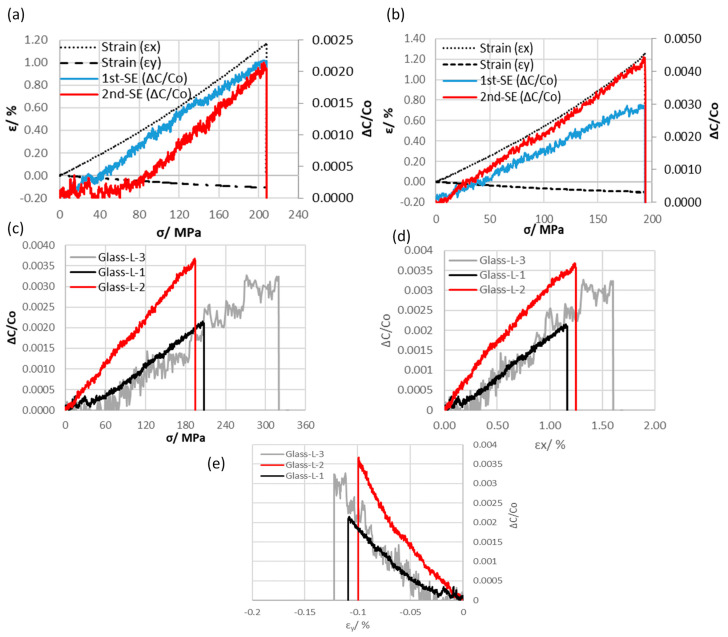
A graph illustrating the relative change in capacitances (first and second sensing electrodes) and nominal (axial and lateral) strain in relation to stress for lateral sensor configuration specimens of (**a**) Glass-L-1 and (**b**) Glass-L-2. (**c**) Axial stress, (**d**) axial strain, and (**e**) lateral strain curves plotted against the average capacitance values for Glass-L-1, Glass-L-2, and Glass-L-3 specimens.

Both Glass-L-1 and Glass-L-2 specimens exhibited similar electrical and mechanical behavior, as shown in Figure 9a,b. However, differences were observed in the results, including axial gauge factor (*k_x_*) and Young’s modulus, with variations of around 40% and 15%, respectively (results summarized in Table 3). To ensure reproducibility, a new specimen (Glass-L-3) was manufactured and tested. Figure 9c–e display the comparison by plotting axial stress, axial strain, and lateral strain curves against the average capacitance value for specimens Glass-L-3, Glass-L-1, and Glass-L-2. Although slight differences still existed in the mechanical and electrical results compared to Glass-L-1 specimens, with discrepancies of approximately 9% in Young’s modulus and 17% in axial gauge factor (*k_x_*), these variations were attributed to differences in thickness between Glass-L-1 and Glass-L-3 samples as illustrated in Table 1. The examination revealed that the new specimen, Glass-L-3, exhibited inherent noise signals. These could be effectively reduced by employing adequate shielding techniques around the sensor and ensuring proper grounding. Such interventions are essential as they significantly reduce interference from external noise sources, thereby enhancing signal clarity.

The embedded sensor in the lateral direction successfully measured both axial (*ɛ_x_*) and lateral (*ɛ_y_*) strain with a linear relative change in capacitance. To estimate the lateral gauge factor (*k_y_*), Equation (2) was utilized. It was also observed that the linear response of the embedded capacitive sensor is directly proportional to the axial stress up to the point of fracture. Previous research [38] has indicated that loading specimens of woven composites in the axial direction results in the straightening of the longitudinal (axial) fiber bundles, thereby pushing the perpendicular coated fiber bundles (1st-SE and 2nd-SE) toward the neutral layer (layer number 4) where the signal ground (GND) is connected. This indirect sensing approach is expected to reduce the average axial gauge factor to approximately 0.25, as compared to *k_x_* ≈ 1 for the axial sensor configuration. In contrast, the lateral gauge factor (*k_y_*) was relatively high (−2.44) compared to the estimated gauge factor in the axial direction. This increased sensitivity is advantageous for monitoring small strains. The relative change in capacitance increased with the applied load, followed by a rapid reduction in capacitance corresponding to the final fracture. Notably, the embedded lateral sensor was unable to directly detect the onset of failure, in contrast to the axial sensor configuration (Glass-A specimens).

**Table 3:** Mechanical and electrical characterization for lateral sensor configuration with coated glass fibers yarn.

Specimen	Young’s Modulus/GPa	*Co*/pF	Gauge Factor/*k_x_*	Gauge Factor/*k_y_*	Ultimate	
					Stress/MPa	Strain/*ɛ_x_* (%)
Glass-L-1	18	10.5	0.19	−1.98	207.9	1.15
Glass-L-2	15.4	11.8	0.32	−3.03	193.4	1.26
Glass-L-3	19.8	11.4	0.23	−2.32	316	1.6
Average	18	11.23	0.25	−2.44	240	1.33

**Figure 10:**
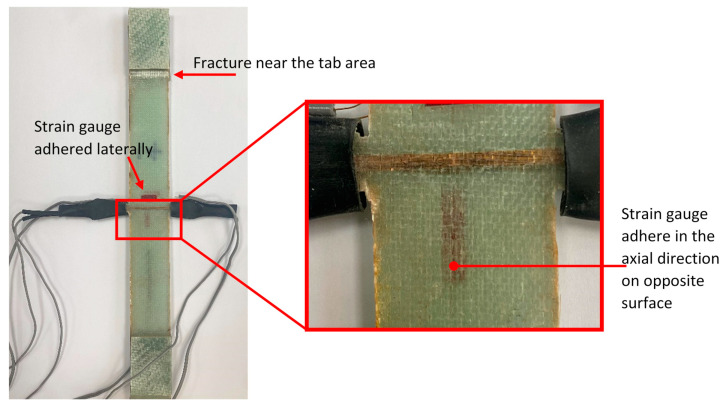
A photograph of a typical fracture in the fiber/epoxy composite specimen. The inset in the photo provides an enlarged view that highlights the area of the integrated sensing electrode that remained intact after the tensile test.

Figure 10 displays a typical fracture for the Glass-L-3 specimen, where a portion of the embedded sensor was visually inspected. The inset in the photo provides an enlarged view that reveals the intact portion of the coated yarn, which remained undamaged after the tensile test. Furthermore, the fracture occurred near the tab, which is relatively distant from the capacitive sensor. As a result, the embedded lateral sensor in the lateral direction cannot directly detect the damage that occurred on the specimen. In contrast to this, the coated yarn configured in the axial direction has the capability to cover the entire composite structure and measure strain along composite layers, enabling the detection of onset damage, as demonstrated in the turning point (see Section 3.2).

## 4. Conclusions and Future Work

In this paper, an in situ structural health monitoring system based on capacitive sensors was developed and implemented for two different composite materials: glass fiber/epoxy composite and Kevlar fiber/epoxy composite. The fabrication process involved several key steps, including the coating of fiber bundles using the physical vapor deposition (PVD) technique, integration of the electrodes, and manufacturing of the composite using a wet lay-up process. The study also investigated the structural characteristics of the coated fibers using scanning electron microscopy (SEM). To assess the functionality of the fabricated electrodes, which were coated with a gold-based material for the differential capacitive system enabling strain monitoring and damage detection, uniaxial tensile tests were conducted. During these tests, the specimens were subjected to tension until they reached fracture, and measurements were collected from the capacitive sensor, recording data on strain and load.

The key findings and conclusions of this study can be summarized as follows:

- Uniform Coating and Low Resistance: The deposition of nanoparticles (Au) using the PVD technique resulted in a uniform coating along the fiber bundles. The coating exhibited strong adhesion to the fibers and had low resistance, measuring only a few hundred ohms for a 1 m length of fibers. This low resistance is essential for forming effective electrodes.
- Axial Sensor Configuration: The integrated capacitive sensor in the axial direction demonstrated linear sensitivity across a wide range of strains. The average gauge factor was approximately 1 for glass fiber/epoxy composite and 1.5 for Kevlar fiber/epoxy composite. This configuration had the capability to cover the entire composite structure and measure strain along composite layers, making it suitable for detecting onset damage that may occur along the sensor’s path.
- Lateral Sensor Configuration: The lateral sensor configuration, which indirectly sensed axial loads, had an average gauge factor of 0.25. However, in the lateral direction, the gauge factor was estimated to be relatively high at around −2.44. This increased sensitivity in the lateral direction makes it suitable for monitoring small strains, but it was less effective in directly detecting damage.

Overall, this study demonstrates the feasibility of using capacitive sensors for in-situ structural health monitoring of composite materials. The choice of sensor configuration (axial or lateral) should be tailored to the specific application and desired sensitivity. The developed system has the potential to enhance the safety and reliability of composite structures by providing real-time strain monitoring and damage detection capabilities. Further research and optimization may be needed to fine-tune the sensor configurations and adapt them to different composite materials and applications.

To further enhance understanding and practical application of embedded capacitive sensors in composite materials, future studies will focus on the continuous assessment of long-term performance under cyclic and fatigue testing conditions. This approach aims to more closely mimic the operational stresses typical of real-world environments, thereby enabling more accurate predictions of the sensors’ durability and lifespan. Additionally, efforts will be directed towards improving the sensitivity of these capacitive sensors to detect minute variations over time, especially in dynamic settings. Such advancements are critical for precise sensor calibration and to ensure consistent and reliable monitoring.

## Figures and Tables

**Table 1:** Main parameters of tensile test specimens.

Sensor Configuration	Type of FRP	Guague Length/mm	Width/mm	Thickness/mm
Axial	Glass-A-1	170	24.92	2.08
	Glass-A-2	170	25.03	1.99
	Kevlar-A-1	170	25.76	2.90
	Kevlar-A-2	170	25.67	2.68
Lateral	Glass-L-1	170	24.85	1.94
	Glass-L-2	170	24.76	1.74
	Glass-L-3	170	24.84	1.72

**Table 2:** Mechanical and electrical characterization for axial sensor configuration with coated glass and Kevlar-aramid fibers.

Specimen	Young’s Modulus/GPa	*Co*/pF	Gauge Factor/k	Failure Initiation Characteristics			Ultimate	
				Stress/MPa	Strain/*ɛ* (%)	$∆C/C_{O}$ (%)	Stress/MPa	Strain/*ɛ* (%)
Glass-A-1	16.8	62.4	1.04	186	0.99	0.94	241.6	1.44
Glass-A-2	17	52	1.00	231	1.38	1.11	272.18	1.60
Average	17	54.2	1.02	208	1.185	1.02	257	1.52
Kevlar-A-1	12.4	37.1	1.45	148.9	1.15	1.25	171.5	1.39
Kevlar-A-2	13.3	37.95	1.57	137.8	0.98	1.07	189.7	1.43
Average	13	37.52	1.51	143.35	1.06	1.16	180.6	1.41

## Data Availability

All data are included in the manuscript, and additional data can be requested from the corresponding author.
